# Effect of *Melilotus* extract on lung injury via the upregulation of tumor necrosis factor-α-induced protein-8-like 2 in septic mice

**DOI:** 10.3892/mmr.2014.2965

**Published:** 2014-11-17

**Authors:** MING-WEI LIU, MEI-XIAN SU, YUN-HUI WANG, CHUAN-YUN QIAN

**Affiliations:** 1 Department of Emergency, The First Hospital Affiliated to Kunming Medical University, Kunming, P.R. China; 2 Surgical Intensive Care Unit, The Second Hospital Affiliated to Kunming Medical University, Kunming, P.R. China

**Keywords:** tumour necrosis factor-α-induced protein-8-like 2, *Melilotus* extract, sepsis, mice, lung injury

## Abstract

As a Traditional Chinese Medicine, *Melilotus* extracts have been reported to function as an anti-inflammatory agent, antioxidant and inhibitor of capillary permeability. The present study aimed to identify the mechanisms by which *Melilotus* interferes with inflammation-associated and oxidative stress pathways during sepsis. An animal model of cecal ligation-perforation (CLP)-induced sepsis was established. Two hours prior to surgery, animals in the treatment group were administered 25 mg/kg *Melilotus* extract tablets and subsequently every 8 h. At 24 h post-administration, pathological modifications in lung tissue and expression levels of tumor necrosis factor-α-induced protein-8-like 2 (TIPE2) expression, nuclear factor (NF)-κB, toll-like receptor 4 (TLR4), heme oxygenase-1 (HO-1), inhibitor of κB kinase (IκB), pro-inflammatory mediators (interleukin-6 and tumor necrosis factor-α), myeloperoxidase (MPO), malondialdehyde (MDA) and superoxide dismutase (SOD), were examined. The results showed that *Melilotus* extract had a marked effect on the pathological manifestation of lung tissue and lung inflammatory response, the upregulation of TIPE2, HO-1 and IκB expression, and the inhibition of TLR4 and NF-κB activities. In addition, following treatment with *Melilotus* extract, the model animals demonstrated decreased levels of MPO and MDA as well as increased levels of SOD. In conclusion, these results indicated that *Melilotus* extract may be a potential therapeutic agent for the treatment of CLP-induced lung injury, the mechanism of which proceeded via inflammation- and oxidation-associated pathways by increasing TIPE2 expression.

## Introduction

Sepsis is a prevalent, severe disease characterized by a systemic inflammatory response to infection (1). It is most apparent in the pulmonary circulation as lungs experience continuous exposure to circulating pathogen-associated molecular patterns, such as endotoxin lipopolysaccharide (LPS), which may initiate an innate immune response (2). Acute lung injury (ALI), characterized by neutrophilic inflammation and pulmonary vascular hyperpermeability, develops in >40% of individuals with sepsis (3). The onset of ALI results in a significant decline in patient prognosis and an increase in intensive care unit mortality from 11 to 38% in septic shock patients (4). Patients that do not succumb to ALI often suffer from long-term morbidity with high healthcare expenditures (5). However, at present, there are no sepsis-specific therapies to prevent the onset of inflammatory lung injury and the underlying mechanisms of septic ALI pathogenesis remain to be fully elucidated.

Tumor necrosis factor-α (TNF-α)-induced protein-8 (TNFAIP8) has important regulatory roles in cell apoptosis, signal transduction, tumor occurrence and development as well as the cell invasion process (6). Numerous studies have focused on the major member of the TNFAIP8 family; TNFAIP8-like 2 (TIPE2) was reported to be necessary for the maintenance of immune homeostasis and was highly expressed in inflammatory tissues, exhibiting negative regulatory effects on the natural immune response (7,8). Previous studies have demonstrated that in the resting state, nuclear factor (NF)-κB within cells exists as a trimer complex composed of two subunits, P65 and P50, and inhibitor of κB kinase (IκB) (9,10). In the absence or mutation of TIPE2, IκB was degraded from the trimer complex by protein kinase C, therefore releasing NF-κB from the cytoplasm and enabling it to translocate to the nucleus. Furthermore, NF-κB, combined with the binding site, initiated the transcription and translation processes of a variety of cytokine genes, including TNF-α, interleukin (IL)-1 and IL-6, therefore inducing the activation of inflammatory cells (11).

Traditional Chinese Medicine and botanical folk medicines, particularly Chinese medicines, which have beneficial effects on fevers and toxicity, were verified to have anti-inflammatory and antioxidant effects by modern pharmacological experiments (12,13). The investigation and application of botanical anti-inflammatory folk medicines which avoid the adverse effects of western medicines is currently a controversial topic, which has been a focus of modern medical studies (14). *Melilotus suaveolens* Ledeb, a type of annual or biennial herbage belonging to the *Melilotus* family of *Leguminosae*, functions to reduce fever, remove toxicity and exert anti-inflammatory effects and detumescence (15). In addition, members of the *Melilotus* family were reported to be applicable to a variety of diseases, including spleen disease, twisted intestinal fever, diphtheria and tonsillitis (16). Several studies have shown that *Melilotus* extract, containing active components including coumarin, flavonoids and tannic acid, functions to inhibit the synthesis and release of inflammatory factors, reduce capillary permeability, improve microcirculation and promote the absorption of edema fluid (17,18,19). *Melilotus* extract tablets have been widely used for clinical purposes; however, they have not been studied in the literature regarding their protection against sepsis-induced lung injury.

The aim of the present study was to determine whether *Melilotus* extract decreased Toll-like receptor (TLR)4 and NF-κB expression via the promotion of TIPE2 expression, which would therefore indicate its protective role against lung injury. Mice with cecal ligation-perforation (CLP)-induced sepsis were used as a model system.

## Materials and methods

### Animals

C57BL/6J (B6) mice were purchased from Kunming Medical University Laboratory Animal Center (Kunming, China). All mice were housed in the Kunming Medical University animal care facility and were maintained in a pathogen-free environment. The mice were aged 8–9 weeks and weighed 20–30 g at the initiation of the experiment, were housed in a vivarium maintained at 23°C with a 12:12 h light/dark cycle (lights off at 7.00pm) and a standard laboratory diet and water were provided *ad libitum*. All experiments were approved by the Ethics Committee of Kunming Medical University (Yunnan, China) and performed according to the guidelines of the Animal Care Committee of Kunming Medical University.

### Reagents

The reverse transcription (RT) reaction kit was obtained from Takara Biotechnology Co. Ltd., (Dalian, China). The polymerase chain reaction (PCR) amplification reagent kit and DNA ladder marker were obtained from Sangon Biological Engineering Co. Ltd (Shanghai, China). β-actin was obtained from Santa Cruz Biotechnology, Inc. (Dallas, TX, USA). TNF-α, IL-6, IL-10 and IL-12 ELISA kits were obtained from Pierce Biotechnology Inc. (Rockford, IL, USA). *Melilotus* extracts were obtained from Seiko Eiyo Yakuhin Co. Ltd (Osaka, Japan).

### Generation of the animal model

C57BL/6J (B6) mice weighing 25–30 g were acclimatized for 1 week following purchase. In order to induce sepsis, mice were anesthetized with isofluorane (4% induction, 2% maintenance; Guangzhou Jin Kang Medical Technology Co., Ltd, Guangzhou, China) and placed on a warming pad (Jinan Ron Trade LLC, Jinan, China). Following laparotomy, the cecum was exteriorized and the membrane between the cecum and mesentery was carefully dissected to release the cecum. The cecum was ligated 4 cm from the tip. Four punctures were made using an 18-gauge needle and 1 mm of faecal material was expressed from the punctures. The incision was sutured in two layers with 4–0 silk. In the sham group, the cecum was located but was not ligated or punctured. Following the procedure, 5 ml warm saline was administered intraperitoneally, the animals were placed on a warming pad and then allowed to recover in individual cages with free access to food and water.

### Generation of TIPE2-deficient mice

TIPE2 genomic fragments of 2.2 and 5.0 kb were amplified using a PCR amplification reagent kit (Sangon Biological Engineering Co., Ltd, Shanghai, China) (20) and cloned, respectively, into the XhoI/NheI and NotI/SalI sites of the pOSDUPDEL vector (a gift from Dr Xiao-Ping Zhong, Department of Pediatrics, Duke University; Durham, NC, USA). TL1 embryonic stem (ES) cells obtained from 129S6/SvEvTac mice were transfected with the targeting vector and subjected to positive and negative selection using G418 (Guangzhou Huowei Chemical Co., Ltd, Guangzhou, China) and ganciclovir (Jena Biosciences, Jena, Germany), respectively. The 129S6/SvEvTac mice, weighing 13–20 g, were purchased from Shanghai Laboratory Animal Center of the Chinese Academy of Science (Shanghai, China), were housed in a temperature-controlled and closed aseptic environment (at a constant temperature of 18–22°C and humidity of 50–80%) under a 12 h light/dark cycle, and provided with free access to sterile water and food. Two ES cell clones were identified using a Southern blot, in which a copy of the TIPE2 gene (including exons 1 and 2) was replaced by the neomycin resistance gene cassette. Mutant ES cells were injected into four-day-old C57BL/6J mouse blastocysts. The resultant chimeric male offspring were crossed with 129S6/SvEvTac females for germline transmission. Unless indicated otherwise, all mice used in the present study were of the 129S6/SvEvTac genetic background. Age- and gender-matched littermates were used as controls.

### Groupings and treatment

Using a random number table, 80 mice were divided into the following four groups: Normal control group, sham-operated group (sham group), sepsis model group (model group) and *Melilotus* treatment group (treatment group), with 20 mice in each group. The model and treatment groups were induced by cecal ligation-perforations (CLP). Animals in the treatment group were administered 25 mg/kg *Melilotus* extract two hours prior to surgery and subsequently every 8 h. The normal control, sham and control groups were administered an identical volume of normal saline. Animals in each group were anesthetized with ether (Wei Sheng Chemical Co., Ltd, Nanjing, China) and sacrificed 24 h following surgery, and the right internal carotid artery was isolated. Blood was extracted (1.5 ml) and centrifuged (10,000 × g for 5 min) to collect the supernatant. The blood was then dispensed into two sterile tubes, which were sealed and stored at −20°C until further use. Furthermore, 2 ml peripheral venous blood was extracted and added to the EDTA anticoagulant, and peripheral blood mononuclear cells (PBMCs) were isolated using the Ficoll density gradient centrifugation method (15,000 × g for 5 min) (21) to detect TIPE2.

### RT quantitative PCR (RT-qPCR) analysis

Total RNA was extracted using Gibco^®^ TRIzol (Thermo Fisher Scientific, Waltham, MA, USA) according to the manufacturer’s instructions. RNA samples were electrophoresed in agarose gels (Shanghai Yuanye Biochemicals Ltd. Shanghai, China) and visualized the gel image using Kodak 1D software (Life Technologies, Grand Island, NY, USA), with ethidium bromide (Beijing Xin Hua Luyuan Science and Technology Co., Ltd, Beijing, China) as a quality control. RNA (3 μg) was incubated with reverse transcriptase for 1 h at 37°C to allow for complementary (c)DNA synthesis. Quantitative changes in messenger (m)RNA expression were assessed using the CFX 96 Real-Time PCR Detection System (Bio-Rad Laboratories, Inc., Hercules, CA, USA) and SYBR Green PCR Master Mix (Shanghai Star-Biological Technology Co., Ltd, Shanghai, China). The PCR master mix consisted of 0.5 units of Taq polymerase, 2 μl of each primer and 3 μl of each cDNA sample in a final volume of 20 μl. All amplifications were repeated three times. Primer sequences used for RT-qPCR are shown in Table I. β-actin was used as an endogenous control and each sample was normalized on the basis of its β-actin content. Relative quantification was calculated using the comparative CT method (2^−ΔΔCt^ method: ^ΔΔCt^=^ΔCt^sample - ^ΔCt^reference). Low ΔCT and ΔΔCT values reflect a relatively high volume of gene transcript. Statistical analyses were then performed for 6–15 replicate experimental samples in each set.

### Western blot analysis

Lung tissues and isolated PBMCs were snap-frozen in liquid nitrogen, pulverized and resuspended in ice-cold lysis buffer (Beijing Solarbio Science & Technology Co., Ltd., Beijing, China). Protein concentrations were determined using the Bradford method (22). Lysates were solubilized on ice for 30 min and the particulate mass was removed using centrifugation (15,000 xg) for 15 min at 4°C. Supernatants were analyzed using 10% SDS-PAGE (Beijing Saichi Biological Technology Co., Ltd, Beijing, China). The primary antibodies used included rabbit anti-TIPE2 monoclonal antibody (1:400), rabbit anti-HO-1 monoclonal antibody (1:400), rabbit anti-NF-κB monoclonal antibody (1:400), mouse anti-IκB monoclonal antibody (1:400) and were purchased from Santa Cruz Biotechnology, Inc. The secondary antibodies used were horseradish peroxidase (HRP)-linked goat anti-rabbit immunoglobulin G (IgG) (1:4,000 dilution; Amersham Pharmacia Biotech, Piscataway, NJ, USA) and sheep anti-mouse IgG-HRP (1:8,000 dilution; Amersham Pharmacia Biotech). The blots were visualized by enhanced chemiluminescence (ECL) using a Pierce ECL western blotting substrate (Pierce Biotechnology, Inc.) and a Johnson enhanced chemiluminescence immunoassay analyzer (Shanghai Qian Jin Industrial Co., Ltd, Shanghai, China).

### Myeloperoxidase (MPO) activity determination

MPO activity was determined using an MPO kit purchased from Nanjing Jiancheng Bioengineering Institute (Nanjing, China) and performed according to the manufacturer’s instructions. In brief, frozen lung samples were thawed and homogenized in ice-cold buffer. Homogenates were then centrifuged at 5,000 × g for 10 min and the pellets were suspended in 0.5% hexadecyl trimethyl ammonium bromide (Shanghai Jinshan Jingwei Chemical Co., Ltd., Shanghai, China) in 50 mM phosphate-buffered saline (PBS; pH 6.0; Wuhan Institute of Biological Products Co., Ltd, Wuhan, China) and incubated at 60°C for 2 h. Following additional centrifugation (5,000 × g for 5 min), the supernatants were collected. The protein concentrations were measured using a protein assay kit (A045; Nanjing Jiancheng Bioengineering Institute). In a 96-well plate, 15 μg protein was incubated with 100 μl 3,3*R*,5,5*R*-tetramethylbenzidine [Yuan (Suqian) Biological Technology Co., Ltd, Suqian, China] for 3 min. Subsequently, 100 μl sulfuric acid (1 N) was added and the absorbance was determined using a UV visible spectrophotometer ( UV-9200; Beijing Rayleigh Analytical Instruments Ltd, Beijing, China) at a wavelength of 450 nm. The original MPO value was normalized against the protein content.

### Superoxide dismutase assay (SOD)

SOD activity was estimated as previously described by Kakkar *et al* (23). The reaction mixture contained 0.1 ml phenazine methosulphate (186 μmol; (Shanghai Yuanye Biotechnology Ltd, Shanghai, China)) and 1.2 ml sodium pyrophosphate buffer (0.052 mmol; pH 7.0; Zhengzhou Lanyu Chemical Co., Ltd, Zhengzhou, China). Following centrifugation (1,500 xg for 10 min followed by 10,000 xg for 15 min) of the homogenate, 0.3 ml supernatant was added to the reaction mixture. The enzyme reaction was initiated by the addition of 0.2 ml NADH (780 μmol; Biotium, Hayward, CA, USA) and terminated after 1 min by the addition of 1 ml glacial acetic acid (Baoding City Bai Yun Chemical Co., Ltd, Shanxi, China). The amount of chromogen formed was measured by recording the color intensity at 560 nm. Results were expressed as U/mg protein.

### Quantification of malondialdehyde (MDA) content

MDA quantification was used to determine lipid peroxidation levels, MDA was quantified as thiobarbituric acid reactive substances (TBARS) kit (Xiao Ke Yuan Biological Technology Co., Ltd, Beijing, China) as previously described (24). In brief, weighed samples were homogenized in 1 ml 5% trichloroacetic acid. The samples were centrifuged (1,500 × g for 10 min), and 250 ml supernatant incubated with the same volume of 20 mM thiobarbituric acid for 35 min at 95°C, followed by 10 min at 4°C. The sample fluorescence was read using a spectrophotometric plate reader with an excitation wavelength of 515 nm and emission wavelength of 553 nm.

### Inflammatory cell quantification in bronchoalveolar lavage fluid (BAL)

As previously described (25), BAL analysis was performed by instilling 0.9% NaCl with 0.6 mmol/l ethylenediaminetetraacetic acid (Qingdao Xinben Chemical Co., Ltd, Qingdao, China) into two separate 0.5-ml aliquots. The fluid was recovered by gentle suction and placed on ice for immediate processing. An aliquot of the BAL was processed for total and differential cell counts; the remainder of the lavage fluid was centrifuged (1,500 × g for 10 min) and the supernatant was removed aseptically and stored in individual aliquots at −70°C. Total cell counts in the BAL were determined using a hemocytometer. The number of different inflammatory cells was calculated as the percentage of certain inflammatory cells multiplied by the total number of cells in the BAL sample. All analyses were performed in a blind manner.

### Cytokine analysis

TNF-α, IL-6, IL-1β, IL-10 and IL-12 levels in BAL were determined using commercially available Mouse cytokine-specific Quantikine ELISA kits (Pierce Biotechnology Inc.), according to the manufacturer’s instructions.

### Vascular permeability assessment

The Evans Blue-conjugated albumin (EBA) extravasation assay was performed as previously described (26). Retroorbital injection of 20 mg/kg EBA (HuanYu Biology Technology Co., Ltd, Suzhou, China) was administered to mice 30 min prior to tissue collection. Lungs were perfused free of blood using PBS, blotted dry and then weighed. Lung tissue was homogenized in 1 ml PBS and incubated with 2X formamide (Suqian Xinya Technology Co., Ltd, Suqian, China) at 60°C for 18 h. The homogenate was then centrifuged at 5,000 xg for 30 min. The optical density of the supernatant was measured at 620 nm and 740 nm. The extravasated EBA in the lung homogenate was expressed as mg Evans Blue dye per g lung tissue.

### Albumin concentration of BAL

The albumin content of the BAL supernatants was assessed using an albumin ELISA kit (E91028Mu; Uscn Life Science, Inc., Hubei, China). Absorbance was measured at 450/540 nm using a microplate reader (Infinite 200; Tecan Group, Ltd, Maennedorf, Switzerland).

### Lung wet/dry (W/D) weight ratio

Following sacrificing the mice, the lungs were surgically dissected away from the heart, trachea and primary bronchi. Each lung was blotted dry, weighed and dried to a constant weight by placing the lung specimen in an oven at 70°C for 48 h. The ratio of the wet lung to dry lung was calculated in order to determine the level of lung edema.

### Histology

A section of the right lung was fixed in formalin, embedded in paraffin wax and stained with Mayer’s hematoxylin and eosin (Merck Millipore, Darmstadt, Germany) for histological examination using a Nikon Eclipse E800 microscope (Nikon Corp., Tokyo, Japan) (27).

### Histology scoring system

Lung sections were evaluated and scored independently by two members of the lab trained in histological assessment, with the use of the scoring system described below. For each mouse, three different lobes were examined for the following features: Interstitial edema, hemorrhage and neutrophil infiltration. Each feature was scored as follows: 0, no injury; 1, minimal injury; 2, moderate injury; and 3, severe injury. The sum of these three scores indicated the total for each lobe and the three lobes were averaged to generate an overall ALI pathological score for each mouse, resulting in a minimum score of 0 and a maximum score of 9.

### Statistical analysis

Values are expressed as the mean ± standard deviation. Statistical calculations were performed using GraphPad Prism 5 (GraphPad Software, Inc, San Diego, CA, USA). For comparisons among multiple groups, a one-way or two-way analysis of variance followed by a Bonferroni post-hoc test were performed. Analysis of linear correlation was used to evaluate the correlation between two variances. P<0.05 was considered to indicate a statistically significant difference between values.

## Results

### TIPE2 deficiency increases NF-κB65 expression in septic mice

**Figure 1:**
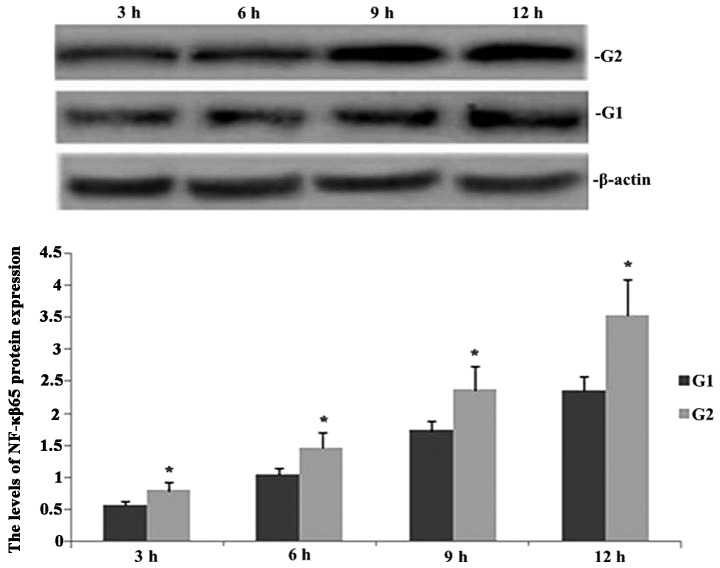
Effect of TIPE2 on NF-κB65 protein expression in septic mice. Western blot analysis was used to determine the protein expression of NF-κB65 at 3, 6, 9 and 12 h following exposure to cecal ligation-perforation. Values are presented as the mean ± standard deviation. ^*^P<0.05 vs. G1. G1, wild-type group, G2, TIPE2^−/−^ group; TIPE2, tumor necrosis factor-α-induced protein-8-like 2; NF-κB65, nuclear factor κB65.

Western blot analysis was performed in order to observe the effect of TIPE2 on NF-κB65 protein in septic mice. As shown in Fig. 1, following CLP surgery, the expression of NF-κB65 was enhanced in TIPE2-/- and wild-type (WT) mice in a time-dependent manner. However, the protein expression of NF-κB65 was significantly increased at each time-point in the TIPE2 deficient mice compared with that of the WT mice.

### Melilotus extracts upregulate TIPE2 and IκB expression and inhibit TLR4 and NF-κB expression

**Figure 2:**
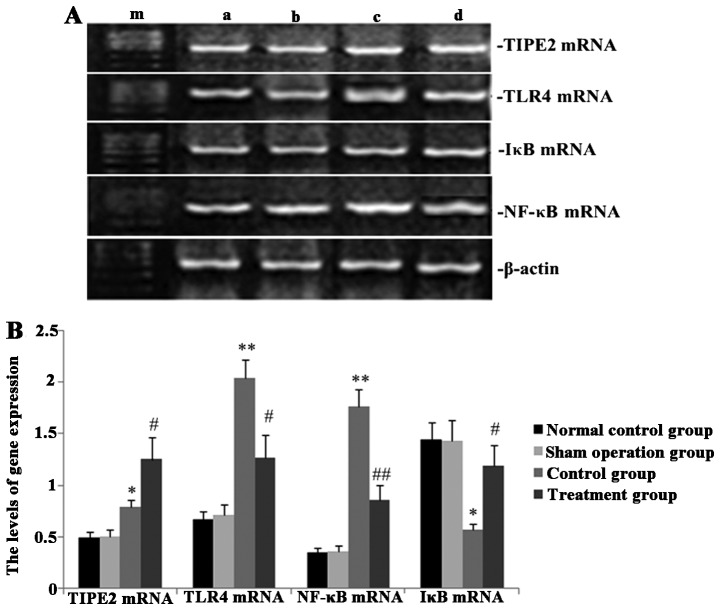
Effect of *Melilotus* extract treatment on TIPE2, TLR4, NF-κB and IκB mRNA expression in septic mice. Reverse transcription-quantitative polymerase chain reaction was used to determine the mRNA expression levels of TIPE2, TLR4, NF-κB and IκB at 24 h post-administration of *Melilotus* extract and exposure to cecal ligation-perforation. (A) Representative image of mRNA expression. Lanes: a, normal control group; b, sham-operated group; c, control group; d, treatment group; m, marker. (B) Quantitative analysis of mRNA expression levels. Values are presented as the mean ± standard deviation. ^*^P<0.05 or ^**^P<0.01 vs. sham-operated and normal control groups, ^#^P<0.05 or ^##^P<0.01 vs. control group. TIPE2, tumour necrosis factor-α-induced protein-8-like 2; NF-κB65, nuclear factor κB65; TLR4, toll-like receptor 4; IκB, inhibitor of κB kinase.

**Figure 3:**
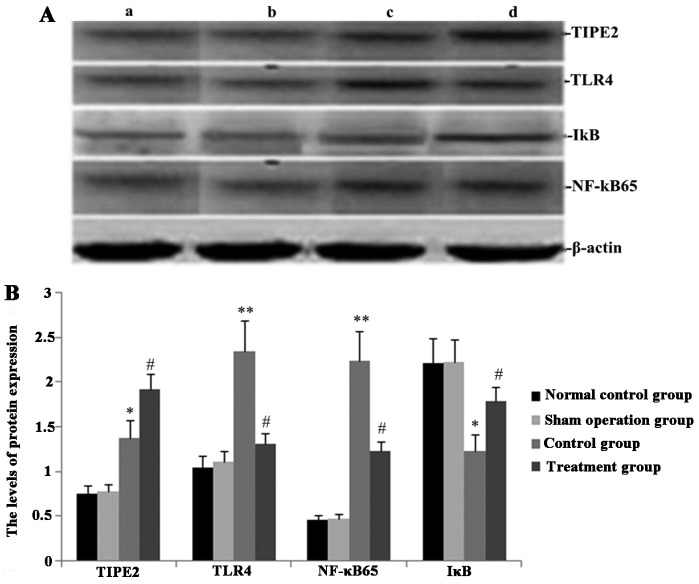
Effect of *Melilotus* extract treatment on TIPE2, TLR4, NF-κB and IκB protein expression in septic mice. Western blot analysis was used to determine the protein expression levels of TIPE2, TLR4, NF-κB and IκB at 24 h post-administration of *Melilotus* extract and exposure to cecal ligation-perforation. (A) Representative western blots of protein expression. Lanes: a, normal control group; b, sham-operated group; c, control group; d, treatment group. (B) Quantitative analysis of protein expression levels. Values are presented as the mean ± standard deviation. ^*^P<0.05 or ^**^P<0.01 vs. sham-operated and normal control groups, ^#^P<0.05 or ^##^P<0.01 vs. control group. TIPE2, tumour necrosis factor-α-induced protein-8-like 2; NF-κB65, nuclear factor κB65; TLR4, toll-like receptor 4; IκB, inhibitor of κB kinase.

RT-qPCR and western blot analyses were performed in order to observe the effect of *Melilotus* extract on TIPE2, TLR4, NF-κB and IκB protein and gene expression in septic mice. As shown in Figs. 2 and 3, following CLP surgery, the mRNA and protein expression levels of TIPE2, TLR4 and NF-κB in the untreated control group were significantly upregulated and IκB expression was downregulated. However, in mice treated with *Melilotus* extract tablets, TIPE2 and IκB mRNA and protein expression levels were significantly upregulated, whereas TLR4 and NF-κB expression was significantly downregulated.

### Melilotus extract decreases proinflammatory cytokine production

**Figure 4:**
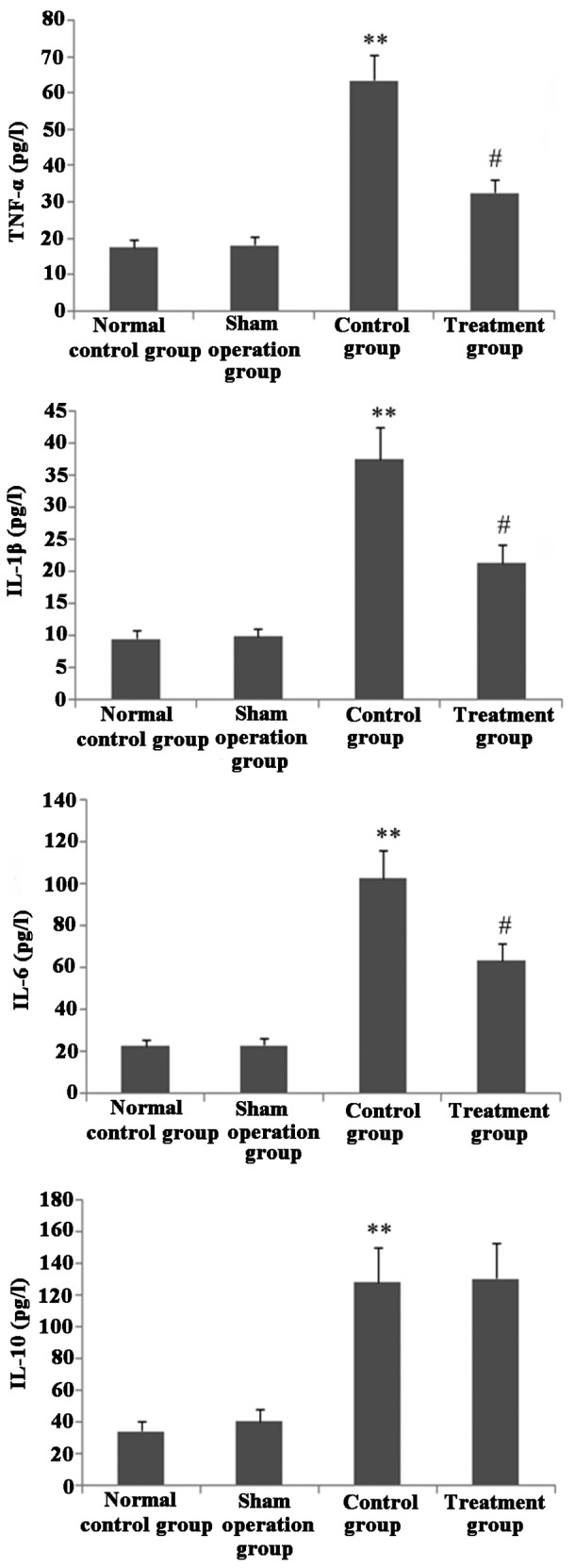
Effect of administration of *Melilotus* extract on TNF-α, IL-6, IL-1β and IL-10 levels in BAL in septic mice. ELISA kits were used to determine the expression levels of TNF-α, IL-6, IL-1β, and IL-10 levels in BAL. Values are presented as the mean ± standard deviation. ^**^P<0.01, vs. sham-operated and normal control groups; ^#^P<0.05 vs. control group. TNF-α, tumor necrosis factor-α; IL, interleukin; BAL, bronchoalveolar lavage fluid.

As shown in Fig. 4, following treatment with *Melilotus* extract, CLP-induced mice exhibited significantly decreased levels of the proinflammatory cytokines TNF-α, IL-1β and IL-6 compared with those in the control group, as determined by BAL. However, the BAL levels of the anti-inflammatory cytokine IL-10 did not significantly change.

### Melilotus extract decreases MPO activity and blocks inflammatory cell infiltration in lung tissue

**Figure 5:**
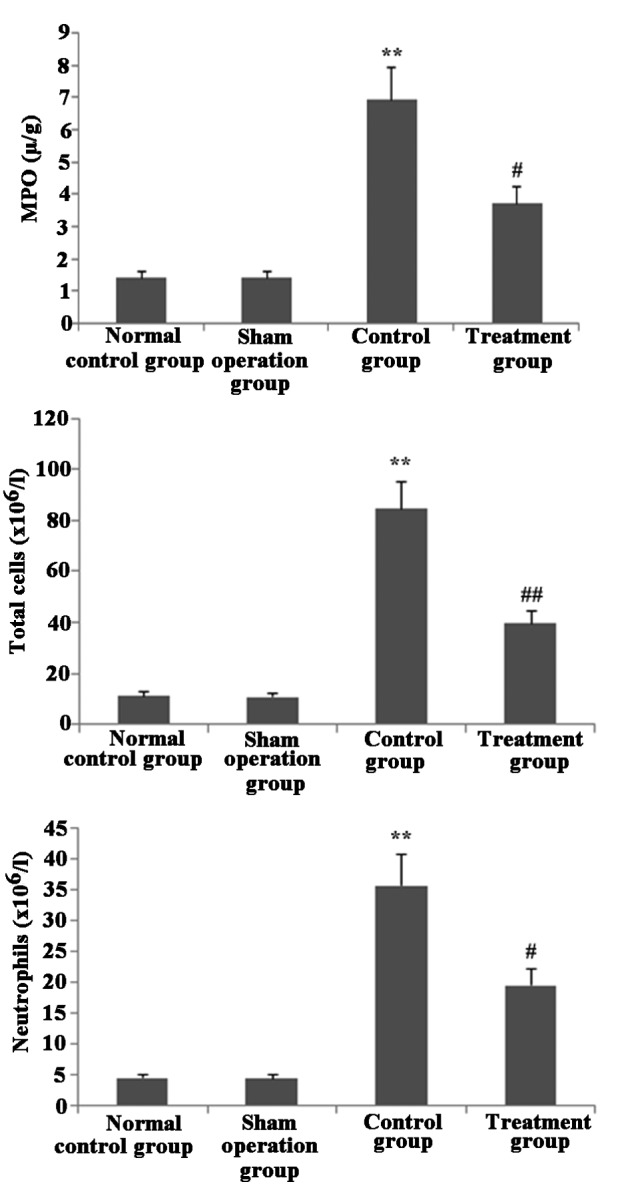
Effect of administration of *Melilotus* extract on MPO activity in lung tissue and inflammatory cells counts in BAL in septic mice. At 24 h following administration of *Melilotus* extract and exposure to cecal ligation-perforation, the MPO activity in lung tissue and inflammatory cell counts in BAL were measured. Values are presented as the mean ± standard deviation. ^**^P<0.01 vs. sham-operated and normal control groups; ^#^P<0.05 or ^##^P<0.01 vs. control group. MPO, myeloperoxidase; BAL, bronchoalveolar lavage fluid.

As shown in Fig. 5, following treatment with *Melilotus* extract, the total numbers of inflammatory cells and neutrophils in BAL and MPO activity in lung tissue were significantly decreased compared with those in the control group.

### Melilotus extract upregulates HO-1 expression, increases SOD activity and prevents MDA activity in lung tissue

**Figure 6:**
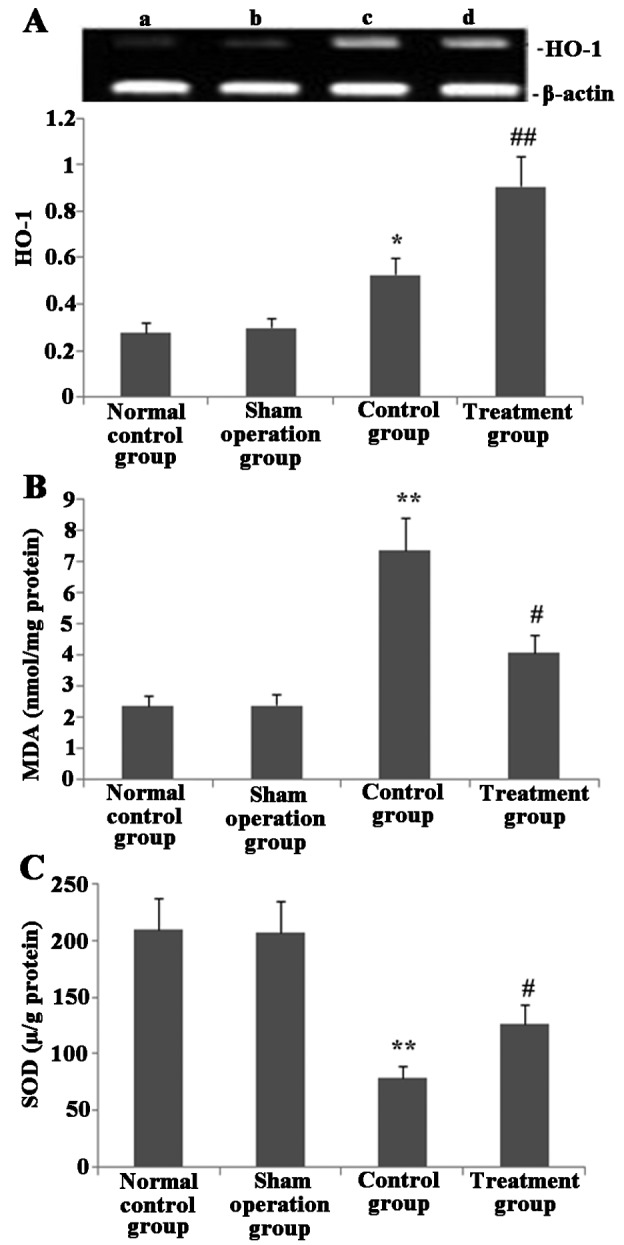
Effect of administration of *Melilotus* extract on HO-1 expression as well as MDA and SOD activities in lung tissue at 24 h following administration of *Melilotus* extract and exposure to cecal ligation-perforation. (A) HO-1 expression: Lanes, a, normal control group; b, sham-operated group; c, control group; d, treatment group. (B) MDA and (C) SOD activities in lung tissue were determined. Values are presented as the mean ± standard deviation. ^*^P<0.05 or ^**^P<0.01 vs. sham-operated and normal control groups; ^#^P<0.05 or ^##^P<0.01 vs. control group. HO-1, heme oxygenase-1; IκB, inhibitor of κB kinase; MDA, malondialdehyde; SOD, superoxide dismutase.

As shown in Fig. 6, 24 h post-administration of *Melilotus* extract and exposure to CLP operation, HO-1 expression and SOD activity in lung tissue were significantly enhanced compared with those in the untreated control group. In addition, the MDA activity in the treatment group was significantly decreased compared with that of the normal control and sham-operated groups.

### Melilotus extract ameliorates lung vascular integrity in septic mice

**Figure 7:**
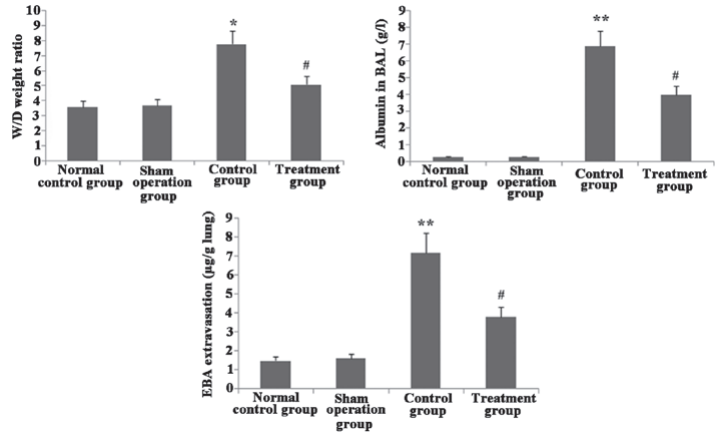
*Melilotus* extract treatment decreases albumin in the BAL, W/D ratio and extravasated EBA in lung tissue in septic mice. At 24 h following administration of *Melilotus* extract and exposure to cecal ligation-perforation. Albumin in BAL, W/D ratio and extravasated EBA in lung tissue were determined and values are presented as the mean ± standard deviation. ^*^P<0.05 or ^**^P<0.01 vs. sham-operated and normal control groups; ^#^P<0.05 vs. control group. BAL, bronchoalveolar lavage fluid; W/D, wet/dry weight ratio; EBA, Evans Blue-conjugated albumin.

As shown in Fig. 7, following CLP surgery, the W/D weight ratio and EBA extravasation in lung tissue as well as albumin in BAL were significantly increased in the untreated control group compared with those in the normal control and sham-operated groups. However, following administration of *Melilotus* extract, there was a significant decrease in the W/D weight ratio, EBA extravasation in lung tissue and albumin in BAL compared to those in the untreated control group.

### Melilotus extract reduces pathological lung injury in CLP-induced mice

**Figure 8:**
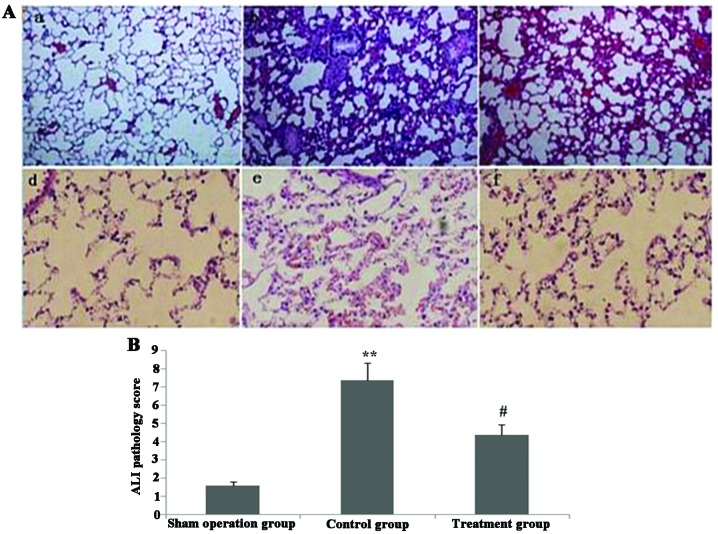
*Melilotus* extract ameliorates the histopathological changes of lung tissue in septic mice. (A) Histopathological changes were determined using hematoxylin and eosin staining in the lung tissue of mice in each of the following groups: a, sham-operated group; b, control group; c, treatment group; d, sham-operated group; e, control group; f, treatment group (magnification: a-c, ×100; d-f, ×400). (B) Quantitative analysis of ALI pathology scores in mice from each group. Values are presented as the mean ± standard deviation (n=3). ^**^P<0.01 vs. sham-operated group, ^#^P<0.05 vs. control group. ALI, acute lung injury.

As shown in Fig. 8, histological analyses of lungs following CLP exposure revealed alveolar septal thickening, accumulation of inflammatory cells in the interstitium and alveoli and an influx of protein-rich fluid into the alveolar space; in addition, ALI pathological score of this untreated CLP surgery group was significantly increased compared with that of the sham-surgery group. However, mice treated with *Melilotus* extracts demonstrated reduced structural changes following CLP exposure and a significantly decreased ALI pathology score compared with that of the untreated group.

## Discussion

In the animal models in the present study, cecal ligation and perforation were used to induce diffused peritonitis in the abdominal cavity by contamination of bacteria present in the intestinal content (28,29), therefore resulting in a wide-range systemic inflammatory response. Mice initially demonstrated hyper-dynamic circulation and hypermetabolism and lower dynamic circulation during a later period, which was consistent with clinical symptoms observed in humans (30).

Medicinal chemistry studies have demonstrated that *Melilotus* plants contain a number of substances which exert anti-inflammatory, antibacterial and antioxidant activities; these substances include coumarin, flavonoids, phenolic acids and saponins. Coumarin constitutes the primary active anti-inflammatory component in *Melilotus* plants (31). Using the erythrocyte sedimentation rate adoption method, Trouillas *et al* (32) investigated the antioxidant properties of water-soluble sites in 16 plant types, including *Melilotus officinalis*. Their results indicated that the antioxidant activities of these plants were positively correlated with the total quantity of phenolic acids. In addition, Parejo *et al* (33) compared the free radical-scavenging activities and antioxidant capacities of 36 different extracts of six plant types, including *Melilotus*, and tested the total phenolic acids using Folin-Ciocalteu colorimetry. The results showed that the extracts of ethyl acetate and dichloromethane contained numerous phenolics, which functioned to scavenge free radicals. A previous study by Pabst *et al* (34) suggested that *Melilotus* extracts containing 0.9% coumarin, 0.2% hydroxyl coumarin and certain flavonoids may improve blood circulation across tissues. Furthermore, Kang *et al* (35) demonstrated that the degree of leukocyte inhibition was an indicator for the evaluation of antiinflammatory activity and revealed that 6 mg azukisaponin V isolated from *Melilotus* extract administered to rats resulted in the suppression of leukocyte production. Zhang *et al* (36) performed an inflammatory swelling and granuloma experiment, which verified that different extracts and coumarin inhibited LPS stimulation in RAW264.7 cells in order to generate pro-inflammatory factors, including IL-6, TNF-α, IL-1β and nitric oxide, as well as promoted the production of the anti-inflammatory factor IL-10. The results of the present study showed that *Melilotus officinalis* enhanced TIPE2 expression, suppressed TLR4 and NF-κB expression, reduced the inflammatory response, increased HO-1 expression, prevented oxidative stress as well as significantly alleviated lung injury; this therefore indicated that *Melilotus officinalis* exhibited effective lung protective abilities.

TLR4, a specific LPS transmembrane receptor, initiates the production of proinflammatory cytokines and corresponding immune response following LPS activation (37). LPS combines with LPS-binding protein (LBP) to form the LPS-LBP complex, which then binds with CD14 (mCD14) on the surface of the cell membrane to form a complex. Following depolarization, LPS in the complex interacts with TLR4 to activate LPS signal transduction pathways, which reinforce NF-κB activity (38). NF-κB, a transcription factor which regulates the expression of proinflammatory cytokines and proteins, is activated in response to several extracellular stimuli and oxidative stress. Activated NF-κB enhances the transcription of numerous cytokines, including TNF-α and IL-6 (38), therefore decreasing the time and increasing the quantity of inflammatory factor synthesis in the inflammatory cells (39,40). In the resting state, NF-κB combines with the inhibitory protein IκB to form an inactive complex within the cytoplasm. When this occurs, cells are stimulated by endotoxins, tumor necrosis factors and other extracellular signals, while IκB kinase phosphorylates and decomposes IκB; subsequently, NF-κB rapidly translocates to the nucleus prior to combining with specific IκB sequences, which induces the transcription of TNF-α, IL-6 and other inflammatory factors as well as adhesion molecules, colony stimulating factors, cyclooxygenase 2 and inducible nitric oxide synthase, thus triggering a systematic inflammatory response (39). Furthermore, inhibition of TLR expression through increasing TIPE2 expression may suppress the activation of NF-κB or promote IκB expression, which in turn contributes to the suppression of inflammation mediator production, thereby inhibiting the occurrence and development of ALI in septic mice.

TIPE2 is a recently identified member of the TNFAIP8 family of immune regulators (41). Certain immune-negative regulatory molecules have important effects acute injury or sepsis. TIPE2, which is thought to be necessary to maintain immune homeostasis, was found to be highly expressed in inflammatory tissues (42). Several studies have reported that LPS, via the stimulation of macrophage TIPE2, downregulated multiple signal transduction pathways (11,43,44). TIPE2 cannot directly act on extracellular signal regulating kinase pathways; however, it was shown to inhibit the activation of terminal kinase of c-jun amino- and p38 mitogen-activated protein kinase, therefore weakening the activity of the transcription factor activator proteins (AP) (45) and depleting TIPE2 expression levels. This may result in the enhancement of the NF-κB sequence and phosphorylation of IκB; in concurrence with this, it was demonstrated that TIPE2 suppressed the activation of AP-1 and NF-κB. Furthermore, TIPE2-deficient cells were found to be highly responsive to the activation of TLR and T-cell receptor signals (46); in addition, in the low-dose LPS-induced sepsis model, TIPE2-knockout mice demonstrated clear septic shock responses compared with those of normal WT mice (47). According to serum analysis, decreased TIPE2 expression resulted in continual lymphocyte activation, which promoted Fas expression and lymphocyte apoptosis (48). However, TIPE2 gene defects may result in the increased production of cell factors, including IL-4, IL-6, IL-12 and IFN-γ (47). The present study demonstrated that the promotion of TIPE2 expression by *Melilotus* extract inhibited the expression of NF-κB and TLR4, thereby inhibiting the production of proinflammatory mediators, including TNF-α and IL-6.

Oxidative stress is an indicator of inflammatory processes. Previous studies have demonstrated that oxidative stress and damage were associated with the pathogenesis and severity of ALI (49–51). The production and release of reactive oxygen species (ROS) is a fundamental anti-microbicidal mechanism, by which ROS upregulation induces tissue damage in sepsis and ALI. MDA, as the primary product of lipid peroxidation, is commonly used as an indicator of the degree of oxidative damage in the body (52). SOD is an important enzyme involved in the dismutation of superoxide radicals, which results from cellular oxidative metabolism into hydrogen peroxide and inhibits LPS-induced penetration. HO-1, also named heat shock protein 32, is a microsomal and rate-limiting enzyme, which catalyzes the degradation of heme into biliverdin, iron atoms and carbon monoxide (53). HO-1 and its breakdown products have vital physiological roles in anti-inflammation, anti-oxidation and the regulation of apoptosis (54,55). The results of the present study demonstrated that upregulating TIPE2 expression using *Melilotus* extracts significantly enhanced HO-1 expression, reduced MDA levels and upregulated SOD activity in damaged lung tissue, indicating that the redox environment of the lungs was improved.

ALI and the more severe stage of acute respiratory distress syndrome (ARDS) are induced by a variety of factors within and outside the lung. ALI/ARDS is characterized by progressive dyspnea and refractory hypoxemia; these are acute syndromes induced through excessive inflammatory responses in the body. Endothelial cell damage and dysfunction are important pathological features of ALI/ARDS (56), which manifest as extensive damage of pulmonary vascular endothelial and alveolar epithelial cells as well as increased pulmonary vascular permeability (57). In the present study, CLP-induced sepsis resulted in increased levels of albumin in BAL, W/D ratio and extravasated EBA in lung tissue as well as revealed an increase in pulmonary vascular permeability. However, these effects were all attenuated by treatment with *Melilotus* extracts, indicating that *Melilotus* extracts reduced pulmonary vascular permeability.

ALI is characterized by an intense inflammatory response. This activates a cascade of proinflammatory events that result in leukocyte infiltration into the lung (56). Therefore, in the present study, MPO activity was measured in lung tissue and the number of inflammatory cells in BAL was quantified. As expected, the increased MPO activity in lung tissues and inflammatory cells count in BAL in the untreated control group was significantly inhibited in mice treated with *Melilotus* extract; in addition, the pathological observations revealed that paraquat-induced lung inflammatory changes were extenuated by *Melilotus* extract treatment.

In conclusion, inflammation immune dysfunction has an important role in the pathogenesis of sepsis. The results of the present study showed that the Traditional Chinese Medicine *Melilotus officinalis* inhibited TLR4 and NF-κB expression, enhanced IκB and HO-1 expression and decreased the inflammatory response and oxidative stress via upregulation of TIPE2 expression in CLP-induced lung injury. Furthermore, early *Melilotus officinalis* treatment following CLP was found to have protective effects, which indicated its potential role in the prevention of LPS-induced ALI.

## Figures and Tables

**Table I:** Reverse transcription-quantitative polymerase chain reaction primer sequences for genes used to validate the microarray analysis.

Gene name	Primer sequence	Size (bp)
TIPE2 mRNA	F, 5′-GGGAACATCCAAGGCAAG-3′ R, 5′-AGCTCATCTAGCACCTCACT-3′	195
TLR4 mRNA	F, 5′-CGCTTTCACCTCTGCCTTCACTACAG-3′ R, 5′-ACACTACCACAATAACCTTCGGCTC-3′	270
NF-κB mRNA	F, 5′-GCACGGATGACAGAGGCGTGTATAAGG-3′ R, 5′-GGCGGATGATCTCCTTCTCTCTGTCTG-3′	420
IκB mRNA	F, 5′-TGCTGAGGCACTTCTGAG-3′ R, 5′-CTGTATCCGGGTGCTTGG-3′	42l
β-actin	F, 5′-GATTACTGCTCTGGCTCCTGC-3′ R, 5′-GACTCATCGTACTCCTGCTTGC-3′	190

F, forward; R, reverse; TIPE2, tumour necrosis factor-α-induced protein-8-like 2; TLR4, toll-like receptor 4; NF-κB, nuclear factor κB; TLR4, toll-like receptor 4; IκB, inhibitor of κB kinase.

## Abbreviations

TIPE2: tumor necrosis factor-α-induced protein-8-like 2
NF-κB: nuclear factor κB
IL-6: interleukin-6
TNF-α: tumor necrosis factor-α
LPS: lipopolysaccharide
CLP: cecal ligation and puncture
RT-PCR: reverse transcription polymerase chain reaction
NE: neutrophil elastase
ALI: acute lung injury
BAL: bronchoalveolar lavage fluid
TNFAIP8: tumor necrosis factor-α induced protein-8
TLR4: toll-like receptor 4
HO-1: heme oxygenase-1
IκB: inhibitor of κB kinase
MPO: myeloperoxidase
MDA: malondialdehyde
SOD: superoxide dismutase
